# Hepatitis C Virus Entry: An Intriguingly Complex and Highly Regulated Process

**DOI:** 10.3390/ijms21062091

**Published:** 2020-03-18

**Authors:** Che C. Colpitts, Pei-Ling Tsai, Mirjam B. Zeisel

**Affiliations:** 1 Department of Biomedical and Molecular Sciences, Queen’s University, Kingston, ON K7L 3N6, Canada; che.colpitts@queensu.ca (C.C.C.); 17plt@queensu.ca (P.-L.T.); 2 Cancer Research Center of Lyon (CRCL), UMR Inserm 1052 CNRS 5286 Mixte CLB, Université de Lyon 1 (UCBL1), 69003 Lyon, France

**Keywords:** hepatitis C virus, viral entry, endocytosis, fusion, cell-to-cell transmission, hepatocyte

## Abstract

Hepatitis C virus (HCV) is a major cause of chronic hepatitis and liver disease worldwide. Its tissue and species tropism are largely defined by the viral entry process that is required for subsequent productive viral infection and establishment of chronic infection. This review provides an overview of the viral and host factors involved in HCV entry into hepatocytes, summarizes our understanding of the molecular mechanisms governing this process and highlights the therapeutic potential of host-targeting entry inhibitors.

## 1. Introduction

Recent estimates from the World Health Organization (WHO) indicate that approximately 71 million individuals are infected by the hepatitis C virus (HCV) worldwide [1]. There is no vaccine to prevent HCV infection. Following infection, the majority of individuals will develop chronic hepatitis C that may subsequently lead to liver cirrhosis and cancer. Although chronic hepatitis C can now be cured using direct-acting antivirals (DAAs), the majority of individuals with chronic hepatitis C remain undiagnosed and untreated. Furthermore, a successful antiviral treatment does not prevent reinfection of patients with risk behaviors. The WHO has recently launched a global program to achieve HCV elimination and so far, only a minority of countries have implemented measures aiming at the elimination of HCV infection as a public health threat within the next decade(s) [2]. While increasing the number of diagnosed/treated cases and reducing risky behavior in defined populations will contribute to micro-elimination of HCV, global eradication of HCV remains challenging and will likely require a protective vaccine [3,4]. 

HCV was discovered in 1989 and subsequently classified in the genus Hepacivirus of the *Flaviviridae* family of viruses [5]. This highly variable RNA virus is further classified into six major genotypes that have distinct geographical distributions. The HCV genome encodes a polyprotein that is subsequently processed into three viral structural proteins that form the viral particle and seven non-structural proteins that are essential for viral replication. The structural proteins comprise the envelope glycoproteins E1 and E2 as well as the capsid protein Core. The Core protein and the viral RNA form the nucleocapsid that is surrounded by a lipid envelope decorated with the E1 and E2 glycoproteins, which drive viral entry. In chronically infected patients, HCV particles circulate as “lipo-viro particles” (LVPs), i.e., virions associated with low-density to very low-density lipoprotein (LDL, VLDL) components including apolipoproteins B (apoB) and E (apoE) [6,7,8,9,10]. By shielding the virus from neutralizing antibodies targeting the HCV envelope glycoproteins, the association of HCV with LDL/VLDL components may contribute to viral evasion of host immune defenses. LVPs appear to be dynamic structures and their composition is influenced by factors affecting lipid metabolism [11]. Electron microscopy observation of viral particles recently showed the long-suspected ultrastructure of HCV [12]. In line with the results from mass spectrometry analyses of viral particles [13,14], electron microscopy confirmed that HCV particles are comprised of both viral and host factors [12,15]. The HCV protease NS3 has also been found associated with HCV particles in proteomic studies [14].

Viral entry is the first step of the viral life cycle and a major target for neutralizing antibodies preventing productive infection. Researchers have aimed to identify the HCV receptor(s) and understand the HCV entry process for more than 20 years. Increasing knowledge about the viral life cycle coupled with technological advances have enabled the development of ever more sophisticated model systems, allowing the discovery of key host factors essential for HCV entry, including those responsible for HCV tissue and species tropism (reviewed in [16,17]). Deciphering their essential roles and interplay in HCV entry has led to the identification of targets for entry inhibitors and has provided clues for rational vaccine design (reviewed in [18,19]). This review provides an overview of the viral and host factors involved in HCV entry into hepatocytes and summarizes our current understanding of the molecular mechanisms governing this process. 

## 2. Host Factors Involved in the First Steps of HCV-Hepatocyte Interactions

**Figure 1:**
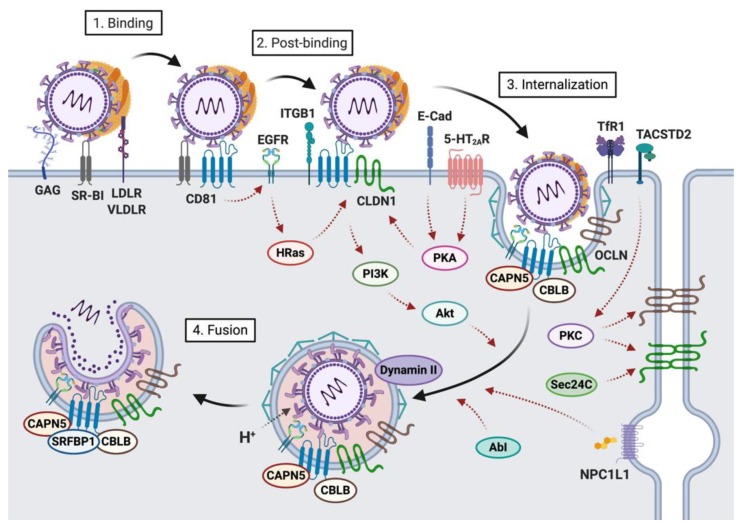
Schematic representation of the cell-free hepatitis C virus (HCV) entry pathway. This cartoon summarizes the host factors and sequence of events leading from initial viral attachment of lipo-viro particles (LVPs) to HCV internalization and release of the viral genome in the cytosol of hepatocytes. The initial binding step primarily involving the lipoprotein component of LVPs likely is a rather unspecific event, which results in the concentration of the virus at the basolateral membrane of hepatocytes and exposure of viral envelope glycoprotein domains that enable the virus to specifically interact with SR-BI, CD81, and CLDN1 (post-binding). The formation of an HCV co-receptor complex is essential for subsequent viral internalization via clathrin-mediated and dynamin-dependent endocytosis. This process is highly regulated by various kinases. Endocytotic vesicles ultimately mature into acidic endosomes, thus promoting low pH-dependent HCV fusion.

The interaction of HCV with hepatocytes leading to viral entry is largely dependent on the interaction of host lipoprotein components and viral envelope glycoproteins with host factors expressed at the hepatocyte surface. Within the past two decades, researchers have identified an abundance of host factors involved in the processes leading from viral attachment to the hepatocyte to receptor-mediated endocytosis of the viral particle and endosomal fusion using various approaches (reviewed in [16,17,20]). Cluster of differentiation 81 (CD81), scavenger receptor class B type I (SR-BI), claudin-1 (CLDN1) and occludin (OCLN) are the four main host factors mediating HCV entry. Indeed, expression of one or several of these host factors can confer cell susceptibility to infection by HCV [21,22,23]. While none of those factors individually confers tissue tropism to HCV, CD81 and OCLN are responsible for the human species-specific tropism of HCV [22,24,25]. In addition to these four essential entry factors, additional host factors play a role in HCV attachment (attachment/binding factors) and internalization/fusion (co-factors). HCV can infect hepatocytes by two distinct routes, i.e., via cell-free virus entry or through cell-to-cell transmission. Summarized below are the host factors and sequence of events leading from initial viral attachment to release of the HCV genome in the cytosol of hepatocytes for the cell-free virus entry pathway (Figure 1). HCV cell-to-cell transmission is described in Section 5.

### 2.1. Host Factors Involved in Viral Attachment to the Hepatocyte Basolateral Membrane

HCV infection occurs via the parenteral route and HCV reaches the liver with the bloodstream. Liver sinusoidal cells may then capture circulating LVPs and facilitate viral infection of neighboring hepatocytes [26,27,28,29]. The liver is the major organ of lipid homeostasis, and hepatocytes express several lipoprotein receptors at their surface. Initial attachment of LVPs to hepatocyte basolateral membranes likely involves virus-associated lipoprotein components (particularly apoE [9,13,30,31,32,33,34,35]), and virus envelope glycoproteins, which interact with highly sulfated heparan sulfate proteoglycans (HSPG) [36,37,38] (particularly syndecans [39,40,41]), LDL receptor (LDLR) [42,43,44,45] and SR-BI [46,47,48,49,50,51] on the cell surface (Figure 1). Interestingly, in addition to their role in viral attachment, these host factors have been shown to also contribute to later steps of the viral life cycle, such as post-binding steps [52,53], internalization [54] or replication [55]. Recently, TIM-1/human hepatitis A virus cellular receptor 1 (HAVCR1)/CD365, a phosphatidylserine receptor that serves as a host factor for various *Flaviviridae* viruses has been identified as an additional factor contributing to HCV attachment via interaction with phosphatidylserine exposed on the HCV envelope [56]. It has been suggested that HCV-TIM-1 interaction may stabilize/enhance viral attachment and promote subsequent interaction with the main entry factors [57]. Given the importance of virus-associated lipoprotein-derived components for the interaction with basolateral hepatocyte membranes, various lipoproteins have been shown to modulate these processes: e.g., high density lipoprotein (HDL) increases HCV pseudoparticle (HCVpp) entry and cell culture-derived HCV (HCVcc) infection while oxidized HDL/LDL inhibit HCVcc infection [58,59,60]. Lipoprotein lipase, which has been reported to function as a bridge between virus-associated lipoproteins and HSPG, can also modulate HCV infection [61,62,63]. Furthermore, a recent study reported that long-chain fatty acyl-coenzyme A can inhibit HCV attachment by targeting virus-associated lipoproteins [64].

### 2.2. Host Factors Involved in Viral Internalization

The initial attachment step primarily involving the lipoprotein component of LVPs likely is a rather unspecific event, which serves to concentrate virions at the basolateral membrane of hepatocytes. It also leads to exposure of viral envelope glycoprotein domains that enable the virus to specifically interact with SR-BI, CD81, and CLDN1 (Figure 1). These three cell surface factors act as viral receptors and contribute to viral entry in a temporally-regulated manner [21,37,52,65,66]. Indeed, they not only directly bind the viral envelope glycoproteins [46,67,68] but also interact with each other [69,70,71,72,73], thereby contributing to the formation of a HCV co-receptor complex that is essential for subsequent viral internalization. Interestingly, several additional host factors that associate with these entry factors have been shown to also contribute to HCV entry (Figure 1), through direct mechanisms or through indirect regulatory mechanisms (see Section 4). 

HCV E2 is first thought to bind to the extracellular loop of SR-BI [46]. The lipid transfer activities of SR-BI [50] may facilitate exposure of binding sites on HCV E2, allowing for the transfer of the viral particle to CD81, which has key roles in subsequent entry steps. SR-BI itself also has a role in post-binding entry steps [53]. Expression levels of SR-BI have been shown to define virus internalization rates, demonstrating the key role of SR-BI in HCV internalization [74,75]. Interestingly, while a splice variant of SR-BI (i.e., SR-BII) has also been demonstrated to promote HCVcc infection, its overexpression did not affect internalization rates, suggesting that SR-BI trafficking plays a role in HCV internalization [74]. High SR-BI surface expression may facilitate the assembly of protein complexes between CD81, SR-BI, and other HCV entry factors to drive internalization [75]. Although initial engagement with SR-BI likely primes E2 for subsequent interactions with CD81, it was recently shown that a small fraction of virions are able to achieve entry in the absence of SR-BI [76].

CD81 is critical for post-binding steps of HCV entry, through its interactions with the HCV E2 glycoprotein [67]. Binding to CD81 activates signaling pathways that promote virion internalization, including activation of receptor tyrosine kinases, such as the epidermal growth factor receptor (EGFR) [77], and Rho and Ras GTPases [71,78]. Activation of EGFR leads to HRas activation [71]. This has been reported to promote actin rearrangements, thus inducing lateral diffusion of CD81 to promote interaction with CLDN1, a protein that is expressed both on the hepatocyte basolateral membrane and at tight junctions. The formation of the CD81-CLDN1 co-receptor complex is a pre-requisite for HCV entry [69,70]. The role of EGFR in regulating the CD81-CLDN1 association is essential for this process [77]. However, EGFR has also been proposed to help recruit clathrin-coated vesicles to aid in HCV internalization [72]. Consistently, EGFR ligands have been shown to enhance the kinetics of HCV entry by inducing the endocytosis of EGFR-CD81 complexes [79]. The kinase MKNK1 has also been suggested to contribute to HCV entry downstream of EGFR [80], although the mechanisms remain to be elucidated. Through these activities, EGFR has a key role in regulating the HCV entry process.

The association of CD81 with the tight junction protein CLDN1 drives HCV internalization [21]. CLDN1 is comprised of four transmembrane domains, with two extracellular loops (EL1 and EL2). Residues within the small highly conserved EL1 of CLDN1 are key for viral entry [21,81], and genetic evidence has suggested a direct interaction between the HCV E1 glycoprotein and CLDN1 [82]. Furthermore, it has also been reported that E1-E2 complexes can interact CLDN1 EL1, whereas soluble E2 did not [68]. Interestingly, other claudins appear to be capable of mediating HCV entry, in a genotype-dependent manner [83]. CLDN6 and CLDN9 are functional as HCV entry factors for some genotypes [84,85], and CLDN12 was recently implicated in HCV entry as well [86,87,88].

OCLN is another tight junction protein that facilitates HCV uptake at a post-binding step [22], although the mechanisms remain less clear. Like CLDN1, OCLN has four transmembrane domains, with two large extracellular loops (EL1 and EL2). OCLN EL2 has been shown to be essential in mediating HCV entry, possibly through its interactions with the endocytosis-promoting GTPase dynamin II [89]. While OCLN does not appear to interact directly with HCV particles, OCLN acts at a similar step as CLDN1 to enable HCV entry. Multiple lines of evidence suggest that OCLN is critical for a late, post-binding entry step [90,91]. 

Alternative entry routes have also been suggested. For example, it has been reported that the VLDL receptor that mediates lipoprotein uptake into hepatocytes might enable HCV to enter hepatocytes *in vivo* in a CD81-independent manner [92]. Since hepatoma cell lines used to study HCV entry do not express VLDLR under classical culture conditions [92], the role of this host factor has not yet been widely studied *in vitro*. While most of the HCV entry factors described herein were identified in the context of classical hepatoma cell lines, the recent development of more sophisticated systems has allowed the validation of these factors under conditions that more closely mimic the *in vivo* hepatic environment. For example, single particle imaging of polarized hepatoma organoids [72] recently showed that HCV localizes with SR-BI, CD81 and EGFR at the basolateral membrane before actin-dependent trafficking to tight junctions, which for the most part fits nicely with the model proposed from studies in hepatoma cell lines.

Several other factors have been shown to contribute to HCV internalization, although the specific mechanisms still remain unclear. The Abl tyrosine kinase was recently identified as a host factor for HCV entry, acting during clathrin-mediated endocytosis [93]. Similarly, the transferrin receptor 1 (TfR1) plays a role in HCV particle uptake [94], although the mechanisms and significance are still poorly understood. Niemann–Pick C1-like 1 (NPC1L1) cholesterol absorption receptor was also identified as an HCV entry factor and likely contributes to HCV entry through its roles as a cholesterol receptor [95].

Ultimately, interactions with these entry factors promote HCV internalization via clathrin-mediated and dynamin-dependent endocytosis [89,96,97] (Figure 1), although alternative endocytotic pathways may also play a role [98]. Endocytotic vesicles ultimately mature into acidic endosomes, thus promoting low pH-dependent HCV fusion. Low endosomal pH is critical to drive conformational rearrangement of the glycoproteins and exposes the fusion peptide. However, interactions of viral glycoproteins with CD81 are also thought to prime the viral particle for fusion by inducing conformational rearrangements in HCV E1 and E2 [99].

## 3. Viral Determinants of Fusion: E1 and E2 Glycoproteins

The HCV E1 and E2 glycoproteins form a noncovalent heterodimer that mediates fusion. Three regions on the E1 and E2 glycoproteins (at positions 270 to 284, 416 to 430, and 600 to 620 on the HCV polyprotein) have been identified to play a role in the membrane fusion process [100], but the fusion mechanism of HCV remains poorly defined. Although HCV was expected to have a class II fusion protein like other *Flaviviridae*, the HCV fusion machinery does not resemble any other known fusion protein, suggesting HCV fusion to be a unique process compared to other fusion mechanisms. Despite harboring a central immunoglobulin (Ig)-fold domain common among class II fusion proteins, the E2 crystal structure revealed a compact globular fold that is inconsistent with the highly-extended class II fusion fold [101,102]. 

Given these disparities with known fusion proteins, it has been proposed that although E2 may mediate HCV entry through interactions with cellular factors, E1 is the HCV fusion protein. Indeed, E1 forms trimers, a feature that is typical for fusion proteins, although E1 trimer formation was dependent on the co-expression of E2 [103]. Furthermore, several studies have identified a hydrophobic region in E1 (CSALYVGDLC) that may represent a putative fusion peptide [104,105,106]. An E1 deletion mutant (lacking residues 268–292) was defective in its ability to form fusion pores and thus rendered HCV pseudoparticles non-infectious [107]. Another recent study identified a central hydrophobic region in E1 that controls requirements for low pH-dependent fusion [108]. However, the N-terminal domain of E1 does not possess the expected class II fusion protein fold [109] and E1 is likely too small to connect viral and cellular membranes [110]. Therefore, HCV fusion is most likely mediated by both E1 and E2, depending on intra- and intermolecular interactions to drive conformational rearrangements of the heterodimer required for fusion. Consistent with this model, interactions between E1 and E2 are critical for entry [68,111] and recent coevolution analysis revealed that E1 and E2 refold interdependently during fusion [112]. In a chaperone-like role, E2 likely supports the fusion properties of E1. The molecular details underlying precisely how the E1-E2 heterodimer mediates membrane fusion still remain to be elucidated.

## 4. HCV Entry into Hepatocytes: A Highly Regulated Process

In contrast to the host entry factors that act as receptors/co-receptors, i.e., via direct interaction with the HCV envelope glycoproteins, others contribute to the regulation of viral entry without directly interacting with viral components, despite being essential for the viral entry process. CD81 and its interacting partners play key roles in regulating entry. Cellular factors that regulate the localization and activity of tight junction proteins also affect HCV entry.

### 4.1. The Regulatory Role of CD81-Tetraspanin Platforms

Several CD81-associated factors have been described [71,73] that indirectly regulate HCV entry. As a tetraspanin CD81 forms large complexes with other membrane proteins and different proteomic approaches identified several CD81-associated factors that have roles in HCV entry, including HRas, integrin β1 (ITGB1), Ras-related protein Rap2B, calpain-5 (CAPN5) and ubiquitin ligase Casitas B-lineage lymphoma proto-oncogene B (CBLB) [71,73]. HRas, through promotion of actin rearrangements, may promote the lateral diffusion of CD81 and its interaction with CLDN1 [78]. Additional CD81 binding partners involved in HCV entry include the serum response factor binding protein 1 (SRFBP1), which is recruited to CD81 during HCV uptake [113] and is thought to act at a late post-binding entry step. Collectively, these data suggest that complex tetraspanin networks may provide platforms for HCV entry and contribute to regulating this process. This is in line with studies having shown that in contrast to hepatocytes, other cell types which are not susceptible to HCV express distinct CD81 partners that restrict HCV entry [114,115,116]. Interestingly, tetraspanin assemblies are emerging as key platforms regulating the entry of unrelated viruses [117,118,119].

### 4.2. Regulation of Tight Junction Proteins

Localization of CLDN1 at the cell surface to facilitate contact with CD81 is critical for HCV entry, and several factors that affect the localization of CLDN1 are important in the HCV entry process (Figure 1). Cell surface localization of CLDN1 (regulated by vesicular transport proteins such as Sec24C) is associated with enhanced HCV entry [120]. Tumor-associated calcium signal transducer 2 (TACSTD2) interacts with CLDN1 and OCLN, and regulates their localization through protein kinase C (PKC)-mediated phosphorylation [121]. A recent study showed that serotonin 2A receptor (5-HT_2A_R) controls CLDN1 localization through protein kinase A (PKA)-mediated phosphorylation [122]. Serotonin receptor 6 (5-HT6) antagonists were similarly shown to mediate CLDN1 localization in a PKA-dependent (yet 5-HT6-independent) manner [123]. These findings are consistent with a pioneering study that demonstrated the importance of PKA for cell surface localization of CLDN1 [124]. E-cadherin is also an important regulator of the cell-surface localization and distribution of CLDN1 and OCLN [125].

## 5. Viral and Host Factors Involved in Viral Cell-to-Cell Transmission

The mechanisms described above are thought to apply to cell-free infection of hepatocytes by LVPs distributed to the liver via the bloodstream. Following this initial hepatocyte infection, HCV is thought to disseminate within the liver using different mechanisms, including cell-free infection following release of newly synthesized viral particles from infected hepatocytes as described above. However, cell-to-cell transmission from an infected hepatocyte to adjacent hepatocytes is critical for viral persistence in the liver [126]. In contrast to cell-free virus entry, HCV cell-to-cell transmission is resistant to the majority of neutralizing antibodies [126,127]. However, this process can be targeted by a variety of entry inhibitors [53,77,128]. Indeed, numerous host entry factors involved in cell-free HCV entry appear to be similarly involved in HCV cell-to-cell transmission. However, since this process has been less extensively studied than cell-free virus entry, the relative contribution of viral and host factors as well as their spatio-temporal interplay remains less characterized. 

Several studies using various approaches have shown that CD81, SR-BI, CLDN1, OCLN, EGFR, ephrin receptor A2 (EphA2), NPC1L1 and LDLR likely contribute to HCV cell-to-cell transmission [41,77,95,126,127]. As for cell-free entry, SR-BI-independent HCV cell-to-cell transmission has been reported [129]. Studies using apoE-silenced donor cells demonstrated that apoE also plays an important role in this process [130,131,132] while apoE expressed by the recipient cells does not appear to be relevant for HCV cell-to-cell transmission [131,132]. Since apoE is required for a late step in the morphogenesis of viral particles and their infectivity [130,133,134], this suggests that mature enveloped viral particles are transferred between adjacent hepatocytes. This is in line with data from a recent reporter-based live-cell visualization study using mutant viruses showing that HCV structural genes and p7 gene are essential for functional HCV cell-to-cell transmission [132]. 

Despite these numerous similarities between cell-free entry and cell-to-cell transmission of HCV, differences in the molecular mechanisms underlying these distinct viral entry pathways have been reported. Indeed, in contrast to cell-free HCV entry that appears to require CD81 (unless hypoxic culture conditions are used [92]), CD81-independent cell-to-cell transmission has been described [135,136]. Furthermore, while apoE/VLDL containing serum has been shown to inhibit cell-free HCV infection, it did not interfere with HCV cell-to-cell transmission [41]. This indicates that although viral and host factors involved in both HCV entry pathways are the same overall, subtle differences in virus-host interactions may exist between both entry routes. Whether these *in vitro* observations have consequences for dissemination of HCV *in vivo* remains to be determined.

Notably, HCV RNA containing exosomes have been reported [137,138,139,140,141]. However, whether and to what extent these exosomes transmit replication competent HCV genomes, proteins and/or virions remains a matter of debate [132,140,142].

## 6. Therapeutic Potential of Host-Targeting Entry Inhibitors

Until the approval of DAA therapy in 2014, viral clearance rates using pegylated interferon α and ribavirin (then the standard-of-care for chronic hepatitis C) were only ~50%. Therefore, researchers were actively investigating alternative antiviral strategies against HCV, including the development of entry inhibitors. As an essential prerequisite for productive viral infection, HCV entry is an attractive antiviral target, with several advantages. Inhibiting viral entry prevents subsequent steps of the viral life cycle and limits viral dissemination. Due to their mechanism of action, entry inhibitors represent an interesting strategy to prevent graft infection in hepatitis C patients undergoing liver transplantation and may also be valuable in the setting of transplantation of organs from HCV positive donors. Entry inhibitors also protect cells from virus-induced modifications and may limit the emergence of resistant variants during viral replication. Of note, entry inhibitors may synergize with DAAs as they act through complementary mechanisms of action [128,143,144]. Entry inhibitors could therefore prove useful in combination therapy regimens. The numerous host factors involved in the HCV entry process offer several possible targets for antiviral intervention. Indeed, several such entry factors—including monoclonal antibodies (mAbs) and small molecule inhibitors—have been evaluated as antiviral targets (reviewed in [18,20,145]) and some have reached preclinical development (Table 1). Their clinical efficacy remains to be demonstrated.

## 7. Conclusions and Perspectives

Given its importance for public health and the limited approaches to manage HCV-infected patients prior to 2014, the field has developed highly relevant model systems and tools to study this virus. As a result of these efforts over the years, HCV entry is now a well-characterized process involving a tremendous array of host factors. Deciphering the essential role played by host factors in viral entry has led to the development of host-targeting entry inhibitors (reviewed in [18,20,145]), a class of antivirals that not only prevent HCV infection, but may also in some cases clear established HCV infection [154]. Increasing knowledge about the viral and host determinants involved in virus-host interactions leading to viral entry also provided valuable information for the understanding of viral escape from neutralizing antibodies and the design of a protective vaccine (reviewed in [160,161], which is a challenge that still remains to be addressed for the global eradication of HCV [3,4]. Furthermore, what has been learned from the study of HCV entry may contribute to understanding the entry pathways of other, less well-characterized, viruses. 

## Figures and Tables

**Table 1:** Host factors involved in HCV entry that have been suggested as antiviral targets. Only host factors for which host-targeting entry inhibitors, e.g., monoclonal antibodies (mAbs) or small molecule inhibitors have at least reached *in vivo* preclinical development are listed. A comprehensive list of host factors and their role(s) in HCV entry is provided in [17].

Host Factor	HCV Entry Steps	Host-Targeting Agents	References
Scavenger receptor BI (SR-BI)	Attachment, postbinding, cell-to-cell transmission	Anti-SR-BI mAbs ITX5061	[46,47,48,49,50,51,52,53,128,146,147,148]
CD81	Postbinding, endocytosis, signaling, cell-cell transmission	Anti-CD81 mAbs	[37,47,67,78,96,149,150,151,152]
Claudin-1 (CLDN1)	Postbinding, endocytosis, cell-cell transmission	Anti-CLDN1 mAbs	[21,66,69,70,96,143,144,153,154]
Occludin (OCLN)	Postbinding, endocytosis, cell-cell transmission	Anti-OCLN mAbs	[22,89,90,155,156,157,158,159]
Epidermal growth factor receptor (EGFR)	Postbinding, endocytosis, signaling, cell-cell transmission	Anti-EGFR mAbs Erlotinib	[77,79]
Niemann-Pick C1-like 1 (NPC1L1)	Postbinding, fusion, cell-cell transmission	Ezetimide	[95]
5-HT_2A_R	Endocytosis, fusion	Phenoxybenzamine	[122]

## Abbreviations

Apo	Apolipoprotein
CAPN5	Calpain-5
CBLB	Casitas B-lineage lymphoma proto-oncogene B
CD81	Cluster of differentiation 81
CLDN	Claudin
DAA	Direct-acting antiviral
EGFR	Epidermal growth factor receptor
EL	Extracellular loop
HCV	Hepatitis C virus
HCVcc	Cell culture-derived HCV
HCVpp	HCV pseudoparticles
HDL	High density lipoprotein
HSPG	Highly sulfated heparan sulfate proteoglycan
LDL	Low density lipoprotein
LDLR	Low density lipoprotein receptor
LVP	Lipoviral particle
mAb	Monoclonal antibody
NPC1L1	Niemann-Pick C1-like 1
OCLN	Occludi
PKA	Protein kinase A
SR-BI	Scavenger receptor class B type I
TfR	Transferrin receptor
VLDL	Very low density lipoprotein
